# A case of pyelonephritis due to ureteral obstruction caused by complete uterine prolapse

**DOI:** 10.1016/j.eucr.2024.102692

**Published:** 2024-02-21

**Authors:** Shohei Tanabe, Kotaro Ichida, Kiyoshi Niiya, Syuji Morishima

**Affiliations:** Kobe City Medical Center, West Hospital, Japan

**Keywords:** Pessary, Pyelonephritis, Uterine prolapse

## Abstract

We present the case of a patient with pyelonephritis secondary to urinary tract obstruction caused by uterine prolapse. An 80-year-old woman with uterine prolapse (pelvic organ prolapse stage 4) was treated with a pessary at an outside hospital due to her high perioperative risk. However, the pessary prolapsed. The patient developed pyelonephritis with hydronephrosis. A pessary was inserted to resolve the blockage of the urinary tract, antibiotic treatment was initiated, and the patient's condition improved. A total vaginal hysterectomy was ultimately performed. Challenges remain in the treatment of pelvic organ prolapse stage 4 for which a pessary cannot be used.

## 1 Introduction

Pelvic organ prolapse (POP) significantly affects the quality of life of patients, with symptoms including urinary, defecatory, and sexual dysfunction. Treatment options include pessaries and surgery; however, asymptomatic patients may be treated conservatively with observation.^1^ POP leads to obstructive uropathy with renal dysfunction that requires treatment with a pessary or ureteral stent. Although cases of pyelonephritis caused by obstructive uropathy resulting from prostate cancer have been reported,^2^ no reports of pyelonephritis due to obstructive uropathy caused by uterine prolapse are available. This report presents the case of a patient with pyelonephritis due to obstructive uropathy caused by uterine prolapse.

## 2 Case presentation

**Fig. 1:**
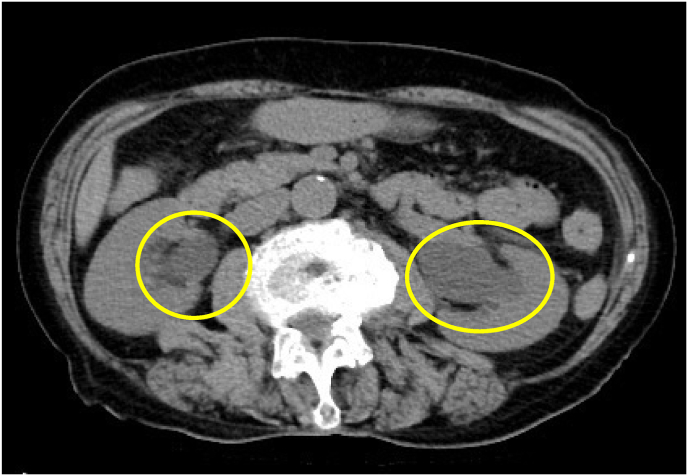
Axial section of CT. Bilateral hydronephrosis is observed on computed tomography (yellow circles). (For interpretation of the references to colour in this figure legend, the reader is referred to the Web version of this article.)

**Fig. 2:**
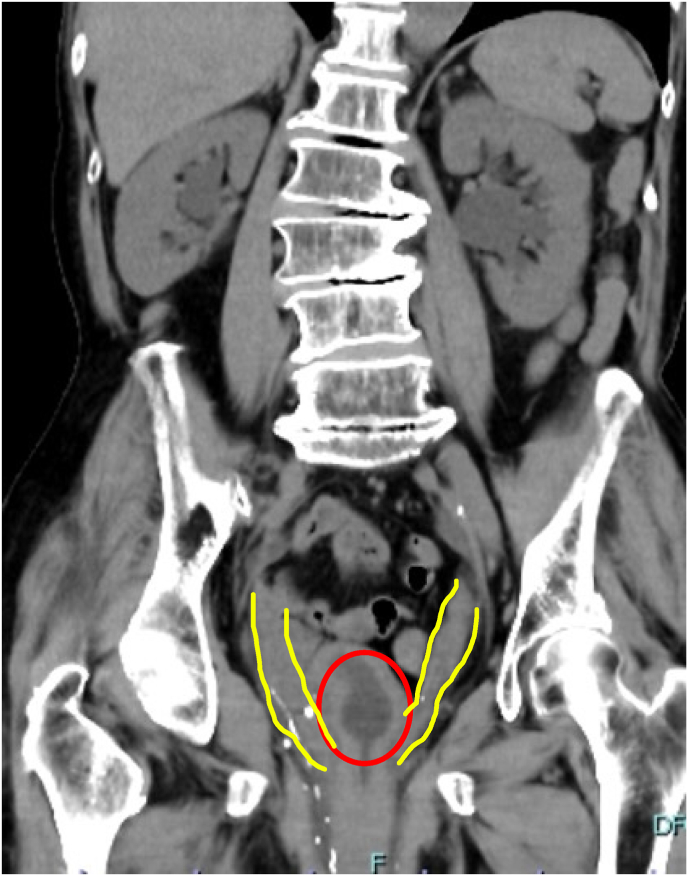
Coronal section of CT. Both ureters are dilated (yellow lines). The balloon in the bladder is apparent on computed tomography (red circle). (For interpretation of the references to colour in this figure legend, the reader is referred to the Web version of this article.)

An 80-year-old woman was referred by her primary care physician for uterine prolapse. She had a history of two pregnancies and vaginal deliveries, numerous cerebral infarctions, and dementia. She was living in an elderly care facility. Upon examination, the patient had complete uterine prolapse (POP stage 4). She also exhibited urinary retention. The patient's regular dose of angiotensin II receptor blockers was continued as it was believed that an anatomical abnormality was the cause of her urinary retention. As the patient was a high surgical risk, conservative treatment was administered, including the insertion of a 56-mm intravaginal ring. However, two days later, the patient returned to the hospital as the ring had prolapsed. A 74-mm intravaginal ring was placed, although it also prolapsed. After consulting the patient and their family, we decided not to use the intravaginal ring and to continue monitoring the patient's clinical progress. Urinary retention did not show improvement. Three days later, the patient developed fever and presented to the emergency department. On arrival, her blood pressure was 86/45 mmHg, her heart rate was 64 beats/min, and her oxygen saturation was 90% on room air. She had an elevated inflammatory response with a white blood cell count of 11,760/μL and a C-reactive protein level of 12.44 mg/dL. In addition, the patient's creatinine level was 2.07 mg/dL and her potassium level was 2.9 mEq/L (Table 1). A urinalysis revealed bacteriuria, and computed tomography (CT) revealed bilateral ureteral dilatation with hydronephrosis (Fig. 1). The patient was diagnosed with pyelonephritis, and blood and urine cultures were collected before the administration of cefmetazole (1 g/day). The patient was also administered potassium. As a ureter was observed below the position of the intravesical balloon on CT (Fig. 2), the urinary tract obstruction was believed to be due to bladder dropout as a result of uterine prolapse. Therefore, an intravesical balloon and a vaginal ring were inserted. Immediately after the insertion of the intravaginal ring, more than 1000 mL of urine was expelled. The next day, the patient's white blood cell count improved to 9150/μL and her creatinine level improved to 1.41 mg/dL. On hospital day 3, the patient's urine culture revealed AmpC beta-lactamase-producing *Escherichia coli*. Therefore, the antibiotics were changed to cefepime 2g/day on hospital day 4. The patient's blood cultures were negative. The intravesical balloon was removed on hospital day 7, and treatment with antibiotics was discontinued on hospital day 8. As the pyelonephritis was secondary to uterine prolapse, surgery was planned on a standby basis. The patient's preoperative creatinine level was 0.81 mg/dL. The obstetrician performed a total vaginal hysterectomy on hospital day 15. The patient had an uncomplicated postoperative course and was discharged to her living facility on postoperative day 11.

**Table 1:** Patient laboratory values.

Fig. 1		
laboratory values		Reference range
Blood values		
white blood cell count (/μl)	11760	3900–9800
Hemoglobin (g/dl)	8.9	11.1–15.1
platelet (/μl)	16600	130000–370000
sodiumu (mmol/l)	133	137–144
potassium (mmol/l)	2.9	3.6–4.8
Creatinine (mg/dl)	2.07	0.47–0.79
Blood urea nitrogen (mg/dl)	38	6.0–22
CRP (ng/dl)	12.44	<0.5

CRP: C-reactive protein.

## 3 Discussion

Bilateral urinary tract obstruction due to uterine prolapse is rare. The patient presented in this report developed pyelonephritis due to urinary tract obstruction, which is even more rare.

The current patient would not have developed pyelonephritis if the initial treatment for uterine prolapse with a pessary had been continued. After the patient developed pyelonephritis, a pessary was placed, her renal function improved, and the pyelonephritis resolved. Previous studies have also reported improved renal function after the treatment of obstructive uropathy with a pessary.^3^

In this patient, a pessary ring was used initially, although it prolapsed outside of the vagina. Intravaginal pessaries and pelvic floor muscle exercises are effective in improving symptoms in patients with POP stages 1–3.^4^ As the patient in this report had POP stage 4, management with a pessary ring and pelvic floor muscle exercises was difficult.

Renal dysfunction can be fully recovered if obstructive urinary tract obstruction lasts less than one week. If the obstruction persists for more than six weeks, the renal function may not recover.^5^ In this patient, the obstruction was resolved early, which may have contributed to an uncomplicated recovery.

## 4 Conclusion

This report describes a case of pyelonephritis caused by urinary tract obstruction due to uterine prolapse. The obstruction was successfully relieved using an intravaginal pessary, leading to a rapid improvement in patient's clinical condition.

The rapid resolution of urinary tract obstruction due to uterine prolapse with a pessary results in favorable outcomes. However, nonsurgical treatment strategies for POP stage 4 uterine prolapse in which an intravaginal pessary cannot be used are limited. Future research should aim to develop additional treatment strategies.

## Funding sources

This study did not receive any specific grants from funding agencies in the public, commercial, or non-profit sectors.

## Informed consent

Informed consent was obtained from the patient presented in this report.

## Declaration of interest

None.

## CRediT authorship contribution statement

**Shohei Tanabe:** Writing – original draft, Supervision. **Kotaro Ichida:** Writing – review & editing. **Kiyoshi Niiya:** Writing – review & editing. **Syuji Morishima:** Writing – review & editing.
